# Thoracic Outlet Syndrome: A Narrative Review

**DOI:** 10.3390/jcm10050962

**Published:** 2021-03-01

**Authors:** Nathan Li, Gregor Dierks, Hayley E. Vervaeke, Allison Jumonville, Alan D. Kaye, Dariusz Myrcik, Antonella Paladini, Giustino Varrassi, Omar Viswanath, Ivan Urits

**Affiliations:** 1 Medical College of Wisconsin–Milwaukee, Milwaukee, WI 53233, USA; linathan006@gmail.com; 2 Louisiana State University Health Sciences Center–Shreveport, Shreveport, LA 71106, USA; gdierk@lsuhsc.edu (G.D.); hverva@lsuhsc.edu (H.E.V.); acamp7@lsuhsc.edu (A.J.); 3 Department of Anesthesiology, Louisiana State University Shreveport, Shreveport, LA 71106, USA; akaye@lsuhsc.edu (A.D.K.); viswanoy@gmail.com (O.V.); ivanurits@gmail.com (I.U.); 4 Department of Internal Medicine, Medical University of Silesia, Katowice, 42-600 Bytom, Poland; dariuszmyrcik@icloud.com; 5 Department of MESVA, University of L’Aquila, 67100 L’Aquila, Italy; antopaladini@gmail.com; 6 Paolo Procacci Foundation, Via Tacito 7, 00193 Roma, Italy; 7 Valley Anesthesiology and Pain Consultants–Envision Physician Services, Phoenix, AZ 85004, USA; 8 Department of Anesthesiology, University of Arizona, Phoenix, AZ 85004, USA; 9 Department of Anesthesiology, Creighton University School of Medicine, Omaha, NE 68114, USA; 10 Southcoast Health, Southcoast Physicians Group Pain Medicine, Wareham, MA 02571, USA

**Keywords:** thoracic outlet syndrome, brachial plexus, subclavian artery, subclavian vein

## Abstract

Thoracic outlet syndrome comprises a group of disorders that result in compression of the brachial plexus and subclavian vessels exiting the thoracic outlet. Symptoms include pain, paresthesia, pallor, and weakness depending upon the compromised structures. While consensus in diagnostic criteria has not yet been established, a thorough patient history, physical exam, and appropriate imaging studies are helpful in diagnosis. General first-line therapy for thoracic outlet syndrome is a conservative treatment, and may include physical therapy, lifestyle modifications, NSAIDs, and injection therapy of botulinum toxin A or steroids. Patients who have failed conservative therapy are considered for surgical decompression. This article aims to review the epidemiology, etiology, relevant anatomy, clinical presentations, diagnosis, and management of thoracic outlet syndrome.

## 1. Introduction

Thoracic outlet syndrome (TOS) comprises a group of disorders that result in compression of the neurovasculature exiting the thoracic outlet and was first described in 1956 [1]. TOS classically occurs in three spaces—the scalene triangle, the costoclavicular space, and the subcoracoid space. Structures involved in TOS include the subclavian artery and vein, the axillary artery and vein, and brachial plexus—any or all of which may be compressed, resulting in distinct clinical pictures, which can include pain, paresthesia, pallor, weakness, feelings of fullness, and muscle atrophy [2,3]. 

TOS is usually subclassified into neurogenic TOS (nTOS), venous TOS (vTOS), and arterial TOS (aTOS), depending on the appropriate etiology upon presentation. TOS can be caused by congenital, acquired, or traumatic factors, although some degree of trauma is usually seen in a majority of TOS cases [4]. Diagnosis depends upon both knowledge of the patient’s existing risk factors as well as their clinical presentation and may be confirmed with physical exam maneuvers, radiographic imaging, or vascular studies.

Treatment of TOS is a multifactorial process, and therapeutic options vary depending on the presenting subtype. While physical therapy is typically the mainstay in conservative nTOS management, other aspects of nTOS treatment may include lifestyle modification, pain management, and anticoagulation [5]. Injection therapy has also been shown to be temporarily effective in reducing symptomatic TOS, as well as a positive surgical prognostic factor [6]. Surgery is usually indicated in symptomatic nTOS candidates who have failed 4–6 weeks of conservative therapy, as well as the vascular etiologies of TOS [7]. While many TOS treatment options exist, the optimal therapy regimen remains unclear. This review aims to provide physicians a brief summary of both pathogenesis, diagnosis, and treatment of TOS, as well as significant findings in the recent literature. A comprehensive electronic literature search, including PubMed, MEDLINE, and Google Scholar databases (1950–2021), was conducted. Previous articles published in peer-reviewed journals, as well as references cited in relevant articles, were also systematically reviewed. Search terms included “thoracic outlet syndrome” AND “diagnosis” OR “imaging” OR “neurogenic” OR “arterial” OR “venous” OR “conservative therapy” OR “injection” OR “surgery”.

This article is based on previously conducted studies and does not contain any studies with human participants or animals performed by any of the authors. Hence, it did not need any approval by the Ethics Committee.

## 2. Epidemiology

The epidemiology of TOS is not firmly established, likely due to a lack of agreement regarding universal diagnostic criteria [8]. The existing data suggest an average incidence of TOS between 3 to 80 cases per 1000 people, typically affecting adolescents to middle-aged adults, especially females between the ages of 20 and 50 [4,9,10]. As mentioned above, TOS is usually subclassified into nTOS, vTOS, and aTOS. nTOS arises from compression of the brachial plexus and comprises the majority of TOS cases, making up for 95% of all diagnosed cases. vTOS and aTOS stem from compression of the subclavian vessels and comprise roughly 4% and 1% of TOS cases, respectively [5]. 

nTOS can be subdivided into true or disputed nTOS, and is categorized as true nTOS with the presence of objective diagnostic abnormalities [11]. True nTOS presents very rarely and is classically a unilateral condition in female patients, while disputed nTOS makes up for 95–99% of all nTOS cases, is often bilateral, and does not feature a classic syndromic vignette as the other types of TOS do [12]. Although disputed nTOS may present with upper extremity symptoms similar to true nTOS, it is also often associated with other diffuse symptoms such as facial pain, visual or hearing disturbances, vertigo, tachycardia, and sleep disturbances [13,14,15].

As mentioned above, vascular TOS may be subdivided into vTOS and aTOS, depending on the compromised structures on presentation. vTOS is often associated with repetitive upper extremity activity and is seen in active young males, with symptoms presenting in the dominant arm [4,12]. Typical symptoms of vTOS include cyanosis in the distal arm, as well as stiffness and tension in the superficial veins of the upper extremity [16,17]. In comparison, aTOS does not follow a gender distribution and is instead often linked to young adults with the presence of certain congenital anomalies such a cervical rib [4]. While aTOS presents the least frequently, it is associated with the most dangerous clinical presentation and usually presents with pain and weakening of the radial pulse with large arm movement, as well as pallor, weakness, and fatigue [13,18,19,20].

As mentioned above, there exists a lack of data regarding the epidemiology and pathology of TOS due to its inconsistent diagnosis. In 2016, the Society for Vascular Surgery released an executive summary regarding new reporting standards for TOS [21]. This document aims to unify terminology used to describe TOS and the diagnostic workup process, as well as improve overall reporting standards in an effort to gather clinical evidence to define better TOS treatment options.

## 3. Etiology

There are many potential causes of TOS, ranging from congenital anomalies to repetitive motion injuries, all of which result in compression of the neurovascular bundle traversing the thoracic outlet. Nearly 70% of cases are related to soft tissue etiologies (such as scalene hypertrophy, regional tumors, or a muscular variation such as the scalenus minimus muscle), with the remaining 30% related to bone abnormalities such as cervical ribs or joint injury with resulting malunion [4,22,23]. 

Additionally, TOS etiologies may be divided into traumatic and nontraumatic causes. A majority of nTOS cases are preceded by neck trauma due to either acute neck hyperextension or midshaft clavicular fracture sustained during motor vehicle accidents [22]. Additionally, fibrosis due to wound healing from acute trauma has also been reported to occasionally compress the neurovasculature, resulting in secondary nTOS [24]. As mentioned previously, muscular hypertrophy can predispose to TOS as well and is an example of a nontraumatic etiology. Chronic poor posture may also result in shortening of the scalene and pectoral muscles and constrict the thoracic outlet [25,26]. Both nTOS and vTOS are occasionally reported in young athletes due to injury from repetitive overhead motions with hypertrophy and fibrosis of the anterior scalene and pectoralis minor muscles [22,27]. Other nontraumatic causes such as the presence of regional tumors and cysts may invade the thoracic outlet and compress the neurovasculature [28]. Cervical ribs are commonly found in aTOS cases, although they are rare and typically asymptomatic in the general population [19].

## 4. Relevant Anatomy

When discussing pertinent TOS anatomy, three spaces of frequent neurovasculature compression are the scalene triangle, costoclavicular space, and subcoracoid space. Table 1 summarizes the borders and relevant contents of each space. The most medial of the three is the scalene triangle, which is bounded anteriorly by the anterior scalene muscle, posteriorly by the middle scalene muscle, and inferiorly by the first rib [29]. The scalene triangle contains the trunks of the brachial plexus and the subclavian artery, while the subclavian vein passes beneath the anterior scalene, avoiding the compartment altogether [30]. Structures within the scalene triangle are often compressed by anatomic variations of the scalene muscles, the presence of the scalenus minimus muscle, or osseous abnormalities such as the presence of a cervical rib [18,19,23,31].

The costoclavicular space lies between the other two spaces and is bounded anteriorly by the clavicle, posteromedially by the first rib, and posterolaterally by the scapula’s upper border [29]. The divisions of the brachial plexus and both of the subclavian vessels traverse this compartment [32]. Compression within this compartment may result from anatomical variation of the subclavian muscle [18,33].

Lastly, the subcoracoid space is bounded anteriorly by the pectoralis minor muscle, posteriorly by ribs 2–4, and superiorly by the coracoid process of the scapula [29]. The subcoracoid space contains the cords of the brachial plexus, the second part of the axillary artery, and the axillary vein, and these contents may be compressed by the clavipectoral fascia or chondrocoracoidal fasciculus [23,24,31].

## 5. Clinical Presentation

The constellation of symptoms seen in true nTOS is caused by compression or irritation of brachial plexus nerves. Pain, paresthesia, and/or weakness in the distribution of affected nerve roots are the hallmarks of nTOS presentation [28]. Compression of the lower plexus (C7-T1) elicits pain of the medial arm, forearm, and hand, paresthesia of the fourth and fifth digits, and hand weakness or loss of dexterity. Similarly, compression of the upper plexus (C5-C7) results in pain in the neck, shoulder, chest, and supraclavicular region, along with arm weakness and paresthesia of the first three digits [34]. In broader terms, patients often complain of proximal pain associated with distal paresthesia or weakness. Reproduction of these symptoms aid in diagnosis and can be accomplished by certain maneuvers (discussed later) or by directly pressing on the affected brachial plexus [3]. Chronic nTOS may also result in thenar, hypothenar, and interossei muscle weakness and atrophy [35]. History of neck trauma should raise suspicion of nTOS, with motor vehicle accidents being the most common inciting incident, followed by stress due to repetitive movement, especially in athletes who practice overhead lifting [3,28]. Additionally, as previously mentioned, disputed nTOS patients may present with similar symptoms of true nTOS, along with a variety of associated symptoms, including facial pain, visual or hearing disturbances, vertigo, tachycardia, and sleep disturbances [13,14,15]. Occipital headaches are another common complaint, occurring in 76% of nTOS patients [28].

Compression of the subclavian vein results in the characteristic symptomatology of vTOS (also called “effort thrombosis” or Paget–Schroetter syndrome), which includes upper extremity swelling, venous engorgement, cyanosis, feelings of arm heaviness, and pain [16,17]. The affected extremity is often noticeably larger than the other, and complaints of pain or heaviness are exacerbated by shoulder abduction. Symptoms are typically acute in onset with the formation of a thrombus in the subclavian vein [16,17,36]. A history of heavy overhead lifting should raise suspicion for vTOS as a chronic venous injury is commonly the inciting cause of venous stenosis followed by thrombosis, and prompt recognition of this clinical presentation can reduce the complications of Paget–Schroetter syndrome [3,32,36].

While aTOS presents the least frequently, it is associated with the most dangerous clinical presentation and may present with pain and weakening of the radial pulse with large arm movement, as well as pallor, weakness, and fatigue. aTOS also commonly presents with hand or upper extremity ischemia due to distal embolization [13,18,19,20]. aTOS is due to clinically evident compression of the subclavian artery and is the least common form of TOS [37]. Although rare, aTOS is the most dangerous subtype of TOS, and patients who present with critical limb ischemia are treated immediately. aTOS patients with chronic limb ischemia should be recognized and treated in an expedited fashion to reduce potentially limb- and life-threatening complications [38,39,40]. Physical exams should aim to identify ischemia and check for a marked discrepancy in blood pressure between the two arms [32]. Classically, aTOS is associated with pallor, pulselessness, pain/paresthesia, and poikilothermia [13,18,19,20,41]. A palpable pulse or audible bruit may be present in the supraclavicular area if an aneurism has formed [32]. As with the other types of TOS, aTOS may be acquired in athletes or occupational lifters. However, it is much more commonly associated with anatomic variances, with an estimated 85% chance of occurring secondary to a cervical rib [32].

## 6. Diagnosis

As mentioned above, nTOS, vTOS, and aTOS each possess a characteristic clinical presentation based on their respective compressed structures [21]. It is, however, important to note that there are often overlaps in symptomatology when multiple structures are compressed. Additionally, although explorative exams are invaluable in confirming the suspected diagnosis, negative results do not rule out TOS [18]. Only a thorough history and physical exam with an emphasis on detecting neurovascular compromise, in combination with additional diagnostic tests and imaging, can accurately diagnose TOS and form an effective treatment plan. 

The history comprises information regarding the onset, evolution, location, quality, intensity, and timing of symptoms, as well as their respective exacerbating and relieving factors [42]. Specific questionnaires used in the workup of pain and disability, such as the McGill Pain Questionnaire or Northwick Park Neck Pain Questionnaire, may also provide invaluable insight into the patient’s symptoms and may be effective in collecting patient history [43,44].

The physical exam is multifaceted and involves an examination of the patient’s posture, cervical alignment, and shoulder blade stability [45]. Additional evaluation of the osseous, muscular, and nervous tissue of the thoracic outlet is usually indicated. The examination consists of careful observation, followed by palpation and various provocative maneuvers, which are discussed in the next section. Bony examination of the thoracic outlet includes the cervical and thoracic spine, first rib, and various joints of the shoulder girdle. Muscular assessment includes evaluation of strength and coordination of the scalene muscles, major and minor pectorals, trapezius, and accessory shoulder girdle muscles. Neurological examinations test the patient’s reflexes as well as sensation [18,42]. Notably, loss of vibratory sense is highly suggestive of TOS, as vibration sensation is quickly lost following compression of the thoracic outlet [43].

### 6.1. Provocative Maneuvers

A hallmark of TOS is the reproducibility of symptoms with specific arm and shoulder movements. Three common physical exam maneuvers used to diagnose TOS include the elevated arm stress test (EAST), the upper limb tension test (ULTT), and the Adson test. These maneuvers are designed to target specific anatomic areas that are commonly implicated in TOS in order to reproduce symptoms of pain, paresthesia, or pulselessness. The maneuvers and positive results are outlined in Table 2. The EAST narrows the costoclavicular space, and subsequent hand gripping may result in pain or paresthesia in the case of nTOS and reduced radial pulse in the case of aTOS [46]. The ULTT stretches the brachial plexus and elicits the symptoms of pain or paresthesia in the setting of TOS [46]. Adson’s test is used to diagnose the compression of structures within the scalene triangle, which is made evident through a diminished radial pulse [35]. Although provocative tests collectively only have an average sensitivity and specificity of 72% and 53%, respectively, they can provide considerable weight to a TOS diagnosis, especially when used in conjunction with imaging techniques [28,42,47].

### 6.2. Imaging and Additional Diagnostic Tests

Although the combination of patient history and detailed physical exam often induce strong clinical suspicion of TOS, imaging is often necessary to confirm both the subtype and exact anatomical site of compression. Plain chest radiographs play a significant role in the diagnosis of all three types of TOS and serve as good initial tests, especially if the clinical presentation is vague [46,48]. Chest X-rays are also useful in identifying bony abnormalities associated with TOS, including cervical ribs, first rib anomalies, focal bone lesions, and congenital malformations [28,48]. CT and MRI also play definitive roles in diagnosing TOS subtypes by providing a more detailed survey of the anatomy [46]. 

In the setting of nTOS, imaging modalities are invaluable in ruling out differential diagnoses such as cervical disc disease, brachial neuritis, or carpal tunnel syndrome. nTOS may also be diagnosed with test injections, electromyography, and nerve conduction studies [21]. However, electromyography and nerve conduction studies are often used in conjunction with other diagnostic tests to rule out other etiologies of neuropathic pain and are rarely utilized alone due to frequent negative, nonspecific, and intermittent findings [3,28]. 

Regarding vTOS and aTOS, duplex ultrasound (DUS) is used to detect subclavian vessel aneurysm, as it is readily available, noninvasive, and can be used during dynamic maneuvers [32,48]. If surgery is not indicated, DUSs may be repeated every six months in asymptomatic aTOS patients for continual monitoring [32]. Venography is considered a mainstay of vTOS diagnosis, and may be combined with intravascular ultrasound to evaluate external vessel compression in vTOS patients with a negative DUS [32]. CT and MR angiography and venography are reliable diagnostic tools that can also identify dynamic changes with positioning and have been deemed appropriate in the diagnosis of aTOS and vTOS by the American College of Radiology [48]. See Table 3 for a summary of imaging studies.

## 7. Conservative Management

Appropriate TOS treatment accounts for factors including disease subtype and etiology as well as severity and duration of symptoms. In the absence of significant vascular compromise, muscle atrophy, or general disability, first-line therapy is most often conservative and comprised of a combination of rest, education, activity modification, physical therapy, and NSAIDs [3,47]. Due to the multifactorial nature of TOS, specific management regimens are selected depending on the patient’s underlying etiology. If the patient’s condition is refractory to a trial of 4–6 months of conservative management, more invasive therapies such as surgery are often considered [7]. 

Broadly, conservative management aims to alleviate neurovascular strain in order to reduce symptom severity and frequency through noninvasive means [49]. Although the optimal regimen of conservative therapy remains unclear, a course of physical therapy combined with applied heat, massage, controlled stretching, acupuncture, NSAIDs, and muscle relaxants have demonstrated reliable efficacy between 59% and 88% of TOS patients, with results lasting over a year after conservative treatment [47,50]. Conservative therapy has shown to be quite efficacious in a majority of patients, and much work has been carried out to identify general prognostic factors for TOS patients participating in conservative therapy. Positive prognostic factors include physical therapy compliance, long-lasting postural and habitual lifestyle modifications, and a sedentary job with few physical demands [51,52,53]. Common negative prognostic indicators of conservative therapy include patient obesity, prior trauma, and the length and severity of TOS symptoms [52,54]. Interestingly, the presence of psychological disorders such as depression has also been correlated with poor patient outcomes in both conservative and surgical treatments [53,55,56,57].

As stated above, physical therapy is particularly effective in relieving symptomatic nTOS patients provided that patients are compliant in completing the prescribed course of therapy. Specific exercises aimed at both strengthening and lengthening postural muscles of the back and shoulder are often prescribed and have demonstrated significant effectiveness in reducing pain [58,59]. 

The benefits of manipulation therapy of the thoracic outlet in the setting of TOS remains unclear. While some studies have shown therapeutic improvements through the manipulative widening of the thoracic outlet, others report worsening of symptoms due to provocation of the neurovascular bundle [45,53]. Instead, techniques that passively elevate and retract the shoulder blade, such as the application of adhesive bandages or braces, have shown promising improvements in pain, paresthesia, upper limb function, and quality of life in patients with moderate to severe symptoms [47,60]. Additionally, education in ergonomics and posture control is essential to aid the patient in avoiding aggravating movements and improve the benefits of physical therapy [7,35].

In addition to standard conservative therapy, mild cases of vTOS and aTOS are treated acutely with intravenous heparin to prevent thrombus expansion and restore blood flow to ischemic areas [3,32,61]. Unfortunately, these subtypes are often refractory to conservative and thrombolytic therapies and require surgical intervention [62].

### Injection Therapy

As previously discussed, structures within the scalene triangle may be compressed by the anterior and middle scalene muscles or the presence of the scalenus minimus muscle [18,19,23,31]. Symptomatic patients with this etiology who fail standard conservative therapy may benefit from nonsurgical muscular decompression through injection of botulinum toxin (BTX-A), trigger point injections, steroids, or anesthetics [6,63].

BTX-A is a neurotoxin that has been historically used to treat pathologic pain due to excessive muscle contraction [64,65]. BTX-A exerts its effect by inhibiting the release of acetylcholine from presynaptic nerve terminals [66,67]. BTX-A may also play a role in directly reducing pain sensation; although this mechanism of action is unclear, studies have shown that it may involve inhibiting the release of nociceptive neuropeptides, including substance P and calcitonin gene-related peptide (CGRP) [68,69]. What is certain is that it reduces the muscular tension pain.

While BTX-A has shown to be effective in the treatment of standard muscle conditions such as spasticity and myofascial pain, the efficacy of BTX-A injections in symptomatic TOS therapy is unclear [63,70,71,72]. Some studies have suggested that BTX-A injections are correlated with significant pain and symptom reduction in 64–69% of TOS patients for up to 3 months [70,71,72]. Additionally, numerous case reports have reported sustained symptom reduction in TOS patients with BTX-A injection therapy [73,74,75]. However, results from a double-blind, randomized, controlled trial did not find any clinically or statistically significant improvements in pain or symptom reduction in TOS patients [63]. Importantly, subjects from this last study suffered a significantly longer duration of symptoms than the control group, with averages of six years and three years, respectively. The increase in the length of symptoms results in a higher risk of central sensitization, with decoupling of painful sensation and peripheral stimuli [76]. Additionally, Finlayson’s study used EMG guidance alone for needle placement, while studies reporting symptomatic improvements through the use of BTX-A injections used fluoroscopic, ultrasound, and CT guidance [71,72,77,78]. Lastly, Finlayson’s study did not set a minimum pain level requirement for participants, resulting in the use of subjects with minimal levels of pain at baseline. Thus, future studies should be conducted to explore the efficacy of BTX-A injections on patients without chronic symptoms, utilize improved imaging methods, and set participant baseline pain level requirements. In total, 90% of successful BTX-A patients experienced significant pain relief following supraclavicular decompression. Therefore, this is a useful indicator of success in surgical decompression [77,79].

In addition to BTX-A and trigger point injections, injection therapy with steroids and local anesthetics including bupivacaine, lidocaine, triamcinolone, and ropivacaine have also been well-documented. They demonstrate good clinical efficacy in TOS treatment, with impressive improvements in paresthesia and overall function when combined with stretching-focused exercise regimens [72,80,81,82,83,84,85]. These findings have been shown to stem from blockage of the muscular elements of the thoracic outlet and not from numbing of the brachial plexus itself [81].

## 8. Surgical Management

Although 60–70% of nTOS cases can be improved with conservative management, some patients still require decompressive surgery [8,61,86,87]. Surgical management of TOS is typically considered after the patient has failed a trial of 4–6 weeks of conservative therapy and presents with worsening symptoms. As mentioned above, refractory cases of vTOS and aTOS also always require surgical management as a majority of cases are due to structural abnormalities with resultant subclavian artery stenosis with or without mural thrombus [32,34,61].

More than half of patients with refractory nTOS and aTOS can be relieved by complete first rib resection and anterior scalenectomy (FRRS) [61,88,89]. The first rib is classically removed via either a transaxillary, supraclavicular, or infraclavicular approach. Although each approach has produced positive outcomes, the transaxillary approach is preferred to the clavicular counterparts in the setting of nTOS due to its rapid and easy exposure of the compressed structures, reducing the risk of injury [61,90,91]. However, the supraclavicular approach is more advantageous in the setting of aTOS, as it allows for easier resection of the first rib, cervical ribs, and other fibromuscular structures which may be compromising the subclavian artery. Additionally, the subclavian artery is often compromised by extensive arterial injury or stenosis in aTOS, and an important advantage of the supraclavicular approach is the ability to address surgically indicated procedures on the subclavian artery. Surgical resections are usually very effective, with significant improvement found in approximately 90% of patients [92]. Surgical patients also reported experiencing greater average levels of improvement than those who only underwent conservative therapy [92].

aTOS is commonly attributed to bony abnormalities such as the presence of a large cervical rib, which may fuse with the first rib and cause subclavian artery compression, resulting in subsequent thrombosis, embolism, and vascular compromise [93]. Surgical management of aTOS consists of three general steps: Firstly, the source of arterial compression is removed. Although FRRS is most commonly performed, it is currently unclear as to whether first rib resection or scalenectomy is more vital for effective aTOS treatment. The first rib is often removed to prevent aTOS recurrence as it is a common fibromuscular anchor point, many of which cause compression of the subclavian artery [94]. However, some authors argue that scalenectomy alone produces similar results utilizing a less invasive procedure, with a lower risk of injury, morbidity, and improved patient recovery times [95,96]. The superiority of either approach remains unclear, and a majority of aTOS patients do well post-FRRS. The second step of aTOS management involves inspection of the subclavian artery for damage due to arterial degeneration, dilation, or aneurysm. If compromised, the artery is repaired with a saphenous vein graft, synthetic prosthesis, or bypass grafting [61,97]. The final consideration in aTOS management involves blood flow restoration in the form of vascular reconstruction. Patients may also require additional embolectomy in concert with vascular reconstruction if there is distal embolization [61].

Current first-line treatment in acute vTOS include thrombolytic therapy and early decompression with FRRS and subclavius tendon division [61,98,99]. Following initial treatment, venography is often used to evaluate the need for venoplasty [8,61,98]. Interestingly, recent studies have suggested that thrombolytic therapy may not reduce the likelihood that a patient will require venoplasty, and thus may not be effective in first-line treatment of vTOS [61,100]. Surgical decompression is also indicated in patients presenting with chronic vTOS, along with preoperative thrombolysis and postoperative anticoagulation to maintain venous patency [32]. Occasionally, vTOS patients will also present with symptoms of intermittent obstruction without accompanying occlusion of the subclavian vein and only require surgical decompression [32]. 

Rib resections carry an inherent risk of neurovascular injury due to poor visualization and incomplete resection [101,102,103]. The use of robotically assisted endoscopic cameras allows for improved visualization of the first rib and the neurovascular bundle, although it is unclear whether this procedure is correlated with stronger symptomatic and postoperative outcomes [101]. Other studies have discussed the advantages of minimally invasive surgical approaches to symptomatic TOS; robotically assisted transthoracic cervical rib resections are associated with improved postoperative wound healing, decreased neurologic complications, and cosmetically favorable scar formation [104,105]. 

## 9. Conclusions

TOS stems from compression of the neurovasculature in the thoracic outlet and can be subdivided into nTOS, vTOS, and aTOS based on the relevant compromised structures and corresponding clinical presentations. The most common cause of TOS is neck trauma, which can arise due to whiplash injuries sustained during a motor vehicle collision. Other populations with increased risk of TOS include athletes who frequently perform overhead movements, patients with tumors or cysts surrounding the thoracic outlet, and those born with anatomic variations such as a cervical or anomalous first ribs. 

Diagnostic criteria for TOS remain controversial, and a thorough history combined with subsequent provocative maneuvers, radiographic imaging, and vascular studies are helpful in elucidating TOS and rule out other differential diagnoses. Early recognition and appropriate treatment are paramount for patient recovery, although this is often difficult due to nonspecific symptoms at initial presentation. First-line therapy is conservative, and most often consists of a combination of physical therapy and pharmacological management. Injection therapy may also be considered if symptoms are due to muscular compression. Surgical intervention is considered only after the patient has failed conservative therapy, although it may be considered earlier if patients present with worsening aTOS or vTOS.

## Figures and Tables

**Table 1:** Anatomical spaces involved in thoracic outlet syndrome.

Space	Borders	Contents
Scalene triangle (i.e. interscalene triangle)	Anterior: Anterior scalene muscle Posterior: Middle scalene muscle Inferior: 1st rib	Brachial plexus (Trunks) Subclavian artery
Costoclavicular space	Anterior: Clavicle/subclavius muscle	Brachial plexus (Divisions)
	Posteromedial: 1st rib	Subclavian artery
	Posterolateral: Upper scapula	Subclavian vein
Subcoracoid space (i.e., retropectoralis minor space)	Anterior: Pectoralis minor muscle	Brachial plexus (Cords)
	Posterior: 2nd–4th ribs	Axillary artery (2nd part)
	Superior: Coracoid process of the scapula	Axillary vein

**Table 2:** Thoracic outlet syndrome diagnostic maneuvers.

Test	Maneuver	Positive Result
Elevated Arm Stress Test (EAST)	Arms are placed in the surrender position with shoulders at a 90° abduction-external rotation and elbows flexed to 90°. Patient maintains this position while slowly opening and closing their hands for 3 min.	Pain, paresthesia, or diminished radial pulse. Pain may prevent patient from continuing the test.
Upper Limb Tension Test (ULTT)	Position 1: Arms abducted to 90° with straight elbows Position 2: Dorsiflexion of wrists Position 3: Tilt head (ear to shoulder) on each side	Positions 1 and 2 elicit symptoms on the ipsilateral side. Position 3 elicits symptoms on the contralateral side.
Adson Test	The affected shoulder is abducted to 30° with full extension of the elbow. Patient extends the neck while turning their head to the ipsilateral shoulder and inhaling deeply.	Diminished or absent radial pulse

**Table 3:** Indicated imaging studies in thoracic outlet syndrome.

TOS Subtype	Always Pertinent	Considerable Studies
nTOS	Chest radiograph	MRI chest with or without contrast CT chest with or without contrast
vTOS	Chest radiograph Duplex ultrasound Catheter venography	CT chest with contrast
aTOS	Chest radiograph Duplex ultrasound Catheter arteriography	CTA chest with contrast MRA chest with and without contrast

## Data Availability

Data available in a publicly accessible repository.
